# Whole cell matrix assisted laser desorption/ionization time-of-flight mass spectrometry (MALDI-TOF MS) for identification of *Leptospira* spp. in Thailand and Lao PDR

**DOI:** 10.1371/journal.pntd.0007232

**Published:** 2019-04-10

**Authors:** Piengchan Sonthayanon, Janthima Jaresitthikunchai, Suthee Mangmee, Tipparat Thiangtrongjit, Vanaporn Wuthiekanun, Premjit Amornchai, Paul Newton, Rattanaphone Phetsouvanh, Nicholas PJ Day, Sittiruk Roytrakul

**Affiliations:** 1 Department of Molecular Tropical Medicine and Genetics, Faculty of Tropical Medicine, Mahidol University, Bangkok, Thailand; 2 Mahidol-Oxford Tropical Medicine Research Unit, Faculty of Tropical Medicine, Mahidol University, Bangkok, Thailand; 3 Proteomics Research Laboratory, National Center for Genetic Engineering and Biotechnology, Pathumthani, Thailand; 4 Lao-Oxford-Mahosot Hospital-Wellcome Trust Research Unit, Microbiology Laboratory, Mahosot Hospital, Vientiane, Lao PDR; 5 Centre for Tropical Medicine & Global Health, Nuffield Department of Clinical Medicine, University of Oxford, Oxford, United Kingdom; Baylor College of Medicine, UNITED STATES

## Abstract

Leptospirosis is a zoonosis with a worldwide distribution, caused by pathogenic spirochetes of the genus *Leptospira*. The classification and identification of leptospires can be conducted by both genotyping and serotyping which are time-consuming and established in few reference laboratories. This study used matrix assisted laser desorption/ionization time-of-flight mass spectrometry (MALDI-TOF MS) as rapid and accurate tool for the identification of leptospires. The whole cell protein spectra of 116 *Leptospira* isolates including 15 references *Leptospira* spp. (pathogenic, n = 8; intermediate, n = 2; non-pathogenic, n = 5) and 101 *Leptospira* spp. clinical isolates was created as an in-house MALDI-TOF MS database. Ninety-seven clinical isolates from Thailand and Laos was validated with these protein spectra and revealed 98.9% correct identification when compared with 16S rRNA gene sequences method. Moreover, MALDI-TOF MS could identify spiked leptospires whole cell in urine. Biomarkers for differentiation of leptospires phylogeny and specific protein spectra for most found *Leptospira* spp. in this area (*L*. *interrogans*, *L*. *kirschneri*, *L*. *borgpetersenii)* based on MALDI-MS algorithm were demonstrated.

## Author summary

Leptospirosis is a world-wide zoonosis caused by pathogenic members of the genus *Leptospira*. Severity of infection ranges from mild features to multi-organ failure and death, although the reason for this is unclear. Clinical symptoms are similar to several other febrile illnesses such as dengue, scrub typhus, malaria, viral hepatitis. Identification of *Leptospira* spp. is generally based on genotyping and serotyping. This study used matrix assisted laser desorption/ionization time-of-flight mass spectrometry (MALDI-TOF MS) for rapid and accurate identification of *Leptospira* whole cell. The methods generated protein spectra of *Leptospira* isolates and created large protein database which can be used to compare and identify other *Leptospira* isolates. The machine learning based on the computational mathematic analysis of this database provide the classification of *Leptospira* phylogeny and biomarkers for species identification.

## Introduction

Leptospirosis is a major public health problem in the developing countries. More than 1 million cases occur worldwide per year, including about 58,900 deaths [1]. In Thailand, prior to 1996, reported cases numbered approximately 200 cases per year, mainly in the central and southern regions. A dramatic increase occurred from 358 cases in 1996 to 14,285 cases in 2000, followed by a continual decline to 2,868 cases in 2005. Reported cases has stabilized at around 2,800–5,500 cases per year during the last 10 years [2]. Although the trend over time is consistent with a sustained outbreak, one of the problems associated with the interpretation of reported cases is that reporting can be done on the basis of a clinical diagnosis alone. Accuracy of clinical diagnosis varied from 0% to 50% between the provinces and was highest during the rainy season [3]. The current laboratory tests available for the diagnosis of leptospirosis are serological testing and molecular-based diagnosis method [4–7]. Matrix-assisted laser desorption/ionization-time of flight mass spectrometry (MALDI-TOF MS) is a novel method that is increasingly used for identification and classification of microorganisms from different genera, species and strains including *Leptospira* spp. [8–10]. The method detects the mass-to-charge ratio of biomolecules from whole cell bacteria and provides spectra within minutes. The protein mass spectra obtained can be added in the MALDI-TOF MS database and used as reference for identification of unknown microorganisms. However, none of known *Leptospira* mass spectra database are commercially available. Here, we employed MALDI-TOF MS to reveal the whole cell protein mass spectra of *Leptospira* spp. including pathogenic, intermediate and saprophytic groups. A large *Leptospira* spp. mass spectra database was established for identification of *Leptospira* spp. from clinical isolates in Thailand and Laos.

## Material and methods

### Bacterial strains

Fifteen references *Leptospira* spp. including pathogenic, intermediate and non-pathogenic groups (Table 1); 101 of *Leptospira* spp. isolates and one isolate of *Turneriella parva*, a spirochete closely related to *Leptospira*, were included in this study (Table 2). *Leptospira* were cultured in Ellinghausen-McCullough-Johnson-Harris liquid medium containing *Leptospira* Medium Base EMJH (Difco, Becton Dickinson, USA), *Leptospira* Enrichment EMJH (Difco) with 3% normal rabbit serum (Invitrogen, USA) at 30°C. All *Leptospira* from human clinical samples were isolated and cultured as described [11]. Serovar identification was performed using cross-agglutination absorption test (CAAT) at WHO/FAO/OIE Collaborating Center for Reference and Research on Leptospirosis, Brisbane, Queensland, Australia. All clinical samples were identified at specie level based on sequencing of 16S rRNA gene as described [12, 13].

**Table 1:** List of 15 references used in this study.

ID no.	Species	Serogroup	Serovar	Strain	Country	Source*	Culture ID	Group**
MD01	*L*. *terpstrae*	Icterohaemorrhagiae	Hualin	LT11-33	China	ATCC	AT1	NP
MD02	*L*. *inadai*	Lyme	Lyme	10	USA	ATCC	AT2	I
MD03	*L*. *alexanderi*	Manhao	Manhao 3	L60	China	NCEZID	AT3	P
MD04	*L*. *borgpetersenii*	Javanica	Javanica	Veldrat Batavia 46	Unknown	ATCC	AT5	P
MD05	*L*. *wolffii*	Khorat	Khorat	H2-Lwof	Thailand	TM-BKK	H2-Lwof	I
MD06	*L*. *alstonii*	Ranarum	Pingchang	80–412	China	KIT	KT01	P
MD07	*L*. *meyeri*	Semaranga	Semaranga	Veldrat Semaranga 173	Indonesia	KIT	KT02	NP
MD08	*L*. *interrogans*	Icterohaemorrhagiae	Lai	Lai	China	WCCRRL	LR31	P
MD09	*L*. *wolbachii*	Codice	Codice	CDC	USA	WCCRRL	LS1	NP
MD10	*L*. *yanagawae*	Semaranga	Sao Paulo	Sao Paulo	Brazil	WCCRRL	LS2	NP
MD11	*L*. *santarosai*	Autumnalis	Alice	Alice	Sri Lanka	WCCRRL	LSS01	P
MD12	*L*. *noguchii*	Autumnalis	Fortbragg	Fort Bragg	United States	WCCRRL	LSS09	P
MD13	*L*. *biflexa*	Semaranga	Patoc	Patoc I	Italy	NIH	LT17	NP
MD14	*L*. *kirschneri*	Cynopteri	Cynopteri	3522 C	Indonesia	NIH	LT29	P
MD15	*L*. *weilii*	Sejroe	Unipertama	K2-1	Indonesia	WCCRRL	we08	P

Abbreviation

*Source

ATCC: American Type Culture Collection, United State of America

NCEZID: NCEZID: National Center for Emerging and Zoonotic Infectious Diseases, Atlanta, USA

TM-BKK: Faculty of Tropical Medicine, Mahidol University, Bangkok Thailand

KIT: KIT Biomedical Research, Amsterdam, Netherlands

WCCRRL: WHO/FAO/OIE Collaborating Centre for Reference & Research on Leptospirosis, Queenland, Australia

NIH: National Institute of Health, Nonthaburi, Thailand.

**Group

NP: non-pathogenic group; P: Pathogenic group and I: Intermediate group.

**Table 2:** List of *Leptospira* isolates (n = 101) and *Turneriella parva* used for *Leptospira* MALDI-TOF MS database.

Bacterial species	Total isolates	Country	Number of isolates	Year	Host	Source*
*L*. *interrogans*	88	Australia	5	1936–1955	Human	WCCRRL
		Belgium	1	1915	Human	WCCRRL
		Czechoslovakia	1	1953	Yellow-throat mouse	WCCRRL
		Denmark	1	1938	Human	NIH
		Indonesia	7	1924–1939	Human (n = 6), bat (n = 1)	WCCRRL; NIH
		Italy	1	1941	Human	WCCRRL
		Jamaica	1	1982	Human	WCCRRL
		Japan	5	1915–1922	Human	WCCRRL; NIH
		Malaysia	8	1953–1966	Human (n = 7), water (n = 1)	WCCRRL; NIH
		Netherlands	1	1931	Dog	NIH
		Papua New Guinea	1	1971	Bandicoot	WCCRRL
		Philippines	2	1957–1959	Rat	WCCRRL
		Russia	1	1966	Long-eared hedgehog	WCCRRL
		Sri Lanka	2	1965–1966	Human	WCCRRL
		Udonthani, Thailand	46	2000–2002	Human	MORU
		Lumpang, Thailand	2	2003	Human	MORU
		Tak, Thailand	1	2004	Human	MORU
		Thailand	1	Unknown	Dog	NIH
* *		Vietnam	1	1967	Human	WCCRRL
*L*. *borgpetersenii*	3	Thailand	3	2002–2003	Human	MORU
*L*. *kirschneri*	5	Russia	1	1928	Human	WCCRRL
		Barbados	1	1985	Dog	WCCRRL
		Bulgaria	1	1951	Human	WCCRRL
		Kenya	1	1968	Unstriped grass rat	WCCRRL
* *		unknown	1	Unknown	Unknown	WCCRRL
*L*. *meyeri*	1	USA	1	Unknown	Frog	NIH
*L*. *noguchii*	1	Unknown	1	Unknown	Unknown	NIH
*L*. *weilii*	3	Laos	1	2009	Human	LOMWRU
		Indonesia	1	1930	Human	NIH
		China	1	1964	Human	WCCRRL
*Turneriella parva*	1	England	1	Unknown	Bacteriological medium	KIT

*Source

WCCRRL: WHO/FAO/OIE Collaborating Centre for Reference & Research on Leptospirosis, Queenland, Australia

NIH: National Institute of Health, Nonthaburi, Thailand

LOMWRU: Lao-Oxford-Mahosot Hospital-Wellcome Trust Research Unit, Lao PDR

MORU: Mahidol-Oxford Tropical Medicine Research Unit, Faculty of Tropical Medicine, Mahidol University, Thailand

KIT: WHO/FAO/OIE and National Leptospirosis Reference centre, KIT Biomedical Research, Amsterdam, Netherlands

### Ethics statement

The studies were approved by the National Ethics Committee for Health Research, Ministry of Public Health of Laos (No 25/NECHR), the Oxford Tropical Ethics Committee, UK and the Ethical Committee of Faculty of Tropical Medicine, Mahidol University, Thailand (Approval no. MUTM-EXMPT 2015–004). Urine sample from healthy donor was provided by the project investigator (Approval no. MUTM2018-054-01).

### Sample preparation for MALDI-TOF Mass spectrometry

*Leptospira* was cultured for a week to obtain 10^8^ colony forming unit (CFU) per ml as previously described [14]. Three milliliters of culture were centrifuged at 13,000 g for 2 min at room temperature and cell pellet was washed with 70% ethanol and centrifuged at 13,000 g for 2 min. The pellet was mixed with 50 μl sinapinic acid matrix solution (Bruker Daltonics, Germany) containing 10 mg of sinapinic acid in 1 ml of 2.5% trifluoroacetic acid (Sigma-Aldrich, USA) and 50% acetonitrile (Sigma-Aldrich, USA). After being well mixed by pipetting and centrifuged at 13,000 g for 1 min, then 2 μl of cell-matrix suspension were added to the ground steel MALDI target plate and dried at room temperature. Each isolates were spotted 24 dots onto the MALDI target plate to test technical replication. Mass spectra were collected using a Ultraflex II MALDI-TOF/TOF mass spectrometer (Bruker Daltonic, USA) operated with FlexControl software in linear positive mode, i.e. using a mass range of 2,000 to 20,000 Daltons. The instrument was externally calibrated with *E*. *coli* strain DH5α ribosomal proteins as recommended protocol.

### Protein spectra database of *Leptospira* spp.

For the 15 reference Leptospiral strains, each individual mass spectrum was analyzed and adjusted for smoothness and baseline using FlexAnalysis software 3.0 (Bruker Daltonics, USA). For each database entry, at least 20 individually measured mass spectra were imported into the MALDI Biotyper 2.0 software. A reference protein main spectra profile (MSP) was calculated and created by following the manufacturer’s recommendations for Ultraflex II measurement and the MALDI Biotyper 2.0 software package. The MSP of 101 *Leptospira* isolates were acquired and added to MALDI Biotyper database for *Leptospira* species identification. The 97 *Leptospira* clinical isolates were blindly identified using Biotyper based-on MSP pattern matching. MSP of each isolates were compared to 101 reference mass spectra in the database and calculated a score value between 0 and 3 reflecting the similarity between isolate and reference spectrum. The results were recorded as identification score and displayed 10-best matching in reference pattern in ranking table. Identification scores ≥ 2.0 were regarded as accepted for a reliable identification at species level and score ≥ 1.7 and ≤ 2.0 for identification at genus level. Scores < 1.7 were considered unreliable as recommended by manufactural.

The reproducibility of MSP was performed. Three reference isolates including *L*. *interrogans*, *L*. *borgpetersenii*, and *L*. *biflexa* were cultured 3 times, MALDI-TOF MS analysed and interpreted using identification score. The MSP reproducibility was also evaluated on different media and different sub-culture times using low passage *L*. *wolffii* from 3 human isolates (strain H8, H9, H16) which had been kept in liquid nitrogen for 14 years. Those isolates were grown in 2 different media (EMJH with 3% normal rabbit serum, EMJH without normal rabbit serum) and 10 sub-culture times. *L*. *wolffii* from each media and culture time were subjected to MALDI-TOF MS after protein extraction. The identification scores were analyzed using ANOVA and student t-tests.

In order to test the detection limit of MALDI-TOF MS method, *L*. *interrogans* MD08 were cultured to reach 10^8^ CFU/ml. After protein extraction, the mixture was diluted to 10-folded serial dilutions to 10 CFU/ml and applied to MALDI-TOF MS.

### MALDI-TOF MS analysis

The statistical analysis was performed using ClinProTools software version 2.2 (Bruker Daltonics, Germany). Twenty spectra of individual isolates were analyzed and visualized for spectral comparison and automatically displayed in a three-dimensional plot based on the Principal Component Analysis (PCA) to discriminate among the analyzed strains.

To discover discriminating peaks or biomarkers specifically for analysis of leptospires phylogeny (n = 51), *Leptospira* species (n = 40), and leptospires serotype (n = 43), four technical replicate spectra of each isolate for training set, as listed in Table 3, were used to generate mathematical models with 3 different algorithms (Genetic Algorithm (GA), Supervised Neural Network (SNN), Quick Classifier (QC)) for classification. The generated classification models were compared based on the calculated Recognition Capacity (RC) and Cross Validation (CV) which indicated the accuracy and reliability of the model. A model with the highest RC and CV was chosen for further peak statistical evaluation. Intensity of peaks presented in the model were evaluated by statistical tests. Peak intensity with *p*-values < 0.05 indicated statistically significant difference. Interesting informative peaks were assessed the capability to discrimination based on Area under the Receiver Operating Characteristic (ROC) curve (AUC) values.

**Table 3:** List of three MALDI-TOF MS training set for identification of biomarkers.

**A. Leptospiral phylogeny analysis**		
**Total number**	***Leptospira* species**	**Number of isolates**
Pathogenic group (n = 39)	*L*. *alexanderi*	1
	*L*. *alstonii*	1
	*L*. *borgpetersenii*	3
	*L*. *interrogans*	23
	*L*. *kirschneri*	6
	*L*. *noguchii*	2
	*L*. *santarosai*	1
	*L*. *weilii*	2
Intermediate group (n = 7)	*L*. *inadai*	1
	*L*. *wolffii*	6
Non-pathogenic group (n = 5)	*L*. *biflexa*	1
	*L*. *meyeri*	1
	*L*. *terpstrae*	1
	*L*. *wolbachii*	1
	*L*. *yanagawee*	1
**B. *Leptospira* species analysis.**		
**Total number**	***Leptospira* species**	**Number of isolates**
Leptospires (n = 40)	*L*. *interrogans*	20
	*L*. *kirschneri*	10
	*L*. *borgpetersenii*	10
**C. Serovar of *L*. *interrogans* analysis.**		
**Total number**	**Serovar**	**Number of isolates**
*L*. *interrogans* (n = 43)	Autumnalis	10
	Bataviae	5
	Canicola	5
	Grippotyphosa	5
	Medanensis	3
	Pomona	5
	Pyrogenes	5
	Sejroe	5

### Detection of spiked *Leptospira* spp. in urine

In order to mimic the detection of leptospires in urine, 4 *Leptospira* spp. including 2 from pathogenic group (*L*. *interrogans* and *L*. *borgpetersenii*), one from the intermediate group (*L*. *wolffii*) and one from the non-pathogenic group (*L*. *biflexa*) were cultured to 10^8^ CFU/ml. Three milliliters of culture were centrifuged at 3,000 g for 10 min, pellet was added with 3 ml urine from healthy donor. After centrifugation at 8,000 g for 10 min., pellet underwent protein extraction. The protein spectra from spiked *Leptospira* in urine were analyzed and compared to the protein spectra acquired from *Leptospira* culture. Urine without spiked *Leptospira* was used as negative control.

## Results

### Identification of protein mass fingerprinting of Leptospires groups

**Fig 1:**
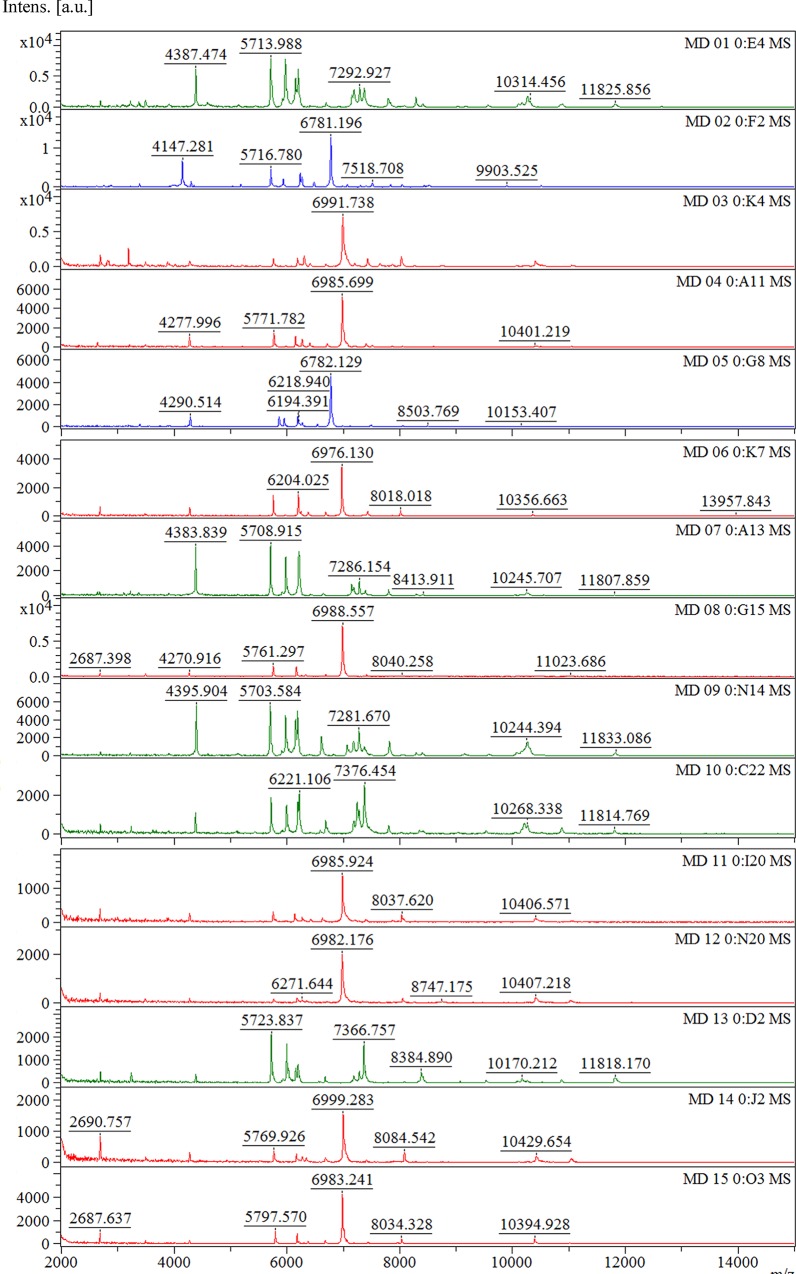
MALDI-TOF protein mass spectra of 15 reference *Leptospira* spp. Protein spectra of pathogenic group (red spectra), intermediate group (blue spectra) and non-pathogenic leptospires (green spectra) are presented. X and Y axis represent mass to charge (m/z) ratio and ion intensity, respectively.

**Fig 2:**
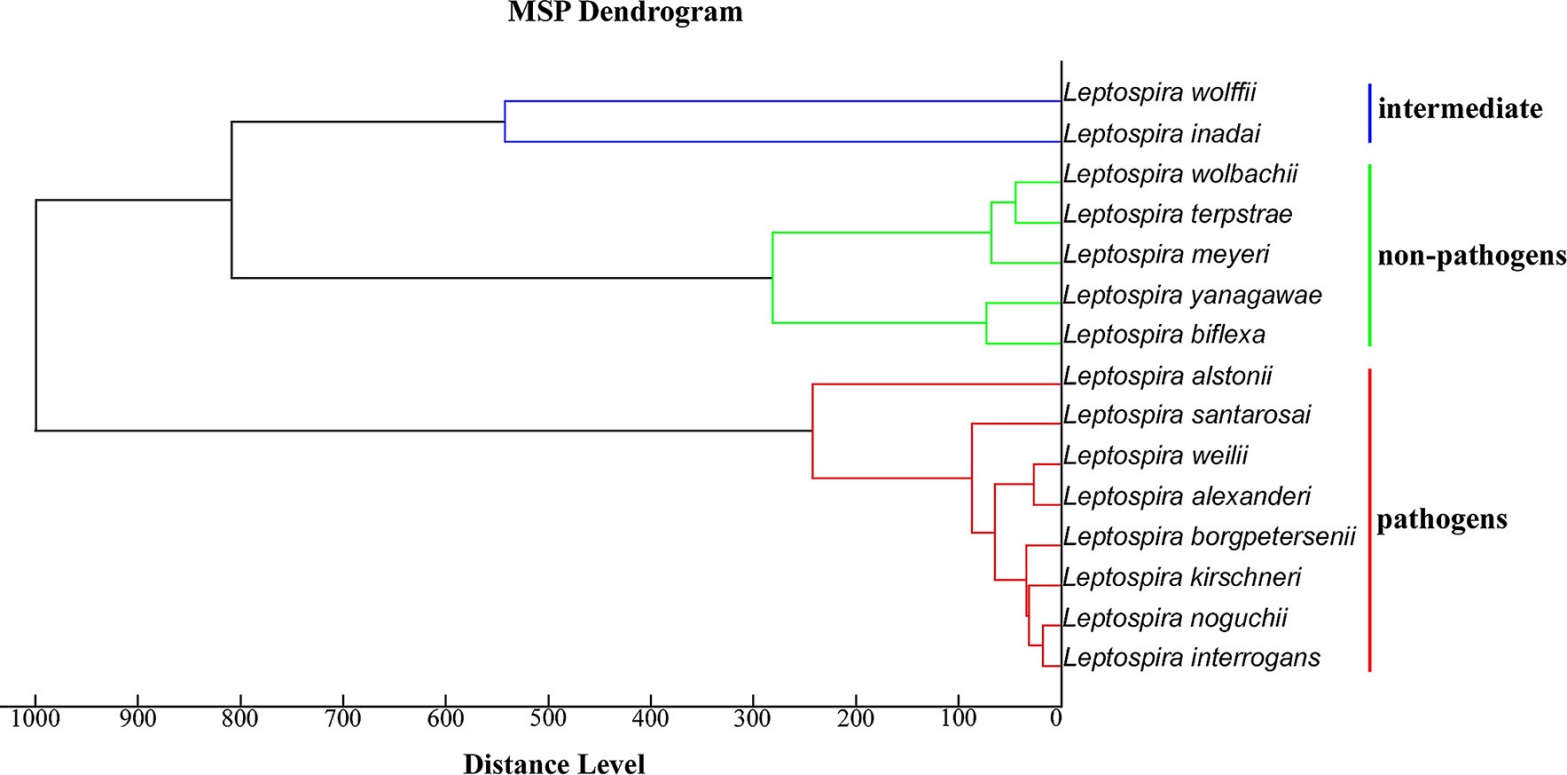
Dendrogram based on main spectrum profile (MSP) of 15 reference *Leptospira* spp. The clustering based on MSP of pathogenic, intermediate and non-pathogenic group were determined.

Fifteen references *Leptospira* spp. (included 8 pathogenic, 2 intermediate and 5 non-pathogenic spp.) generated by MALDI-TOF MS revealed protein profile at mass to charge ratio (m/z) ranged from 2–20 kDa. These protein profiles showed specificity representing individual species (Fig 1). The discrimination of *Leptospira* spectra was demonstrated by differentiating 3 different groups in the MSP dendrogram which related to their phylogeny (pathogenic, intermediate and non-pathogenic groups) (Fig 2). This result indicated that phylogeny of *Leptospira* could be clustered based on their protein profile using MALDI-TOF MS.

**Fig 3:**
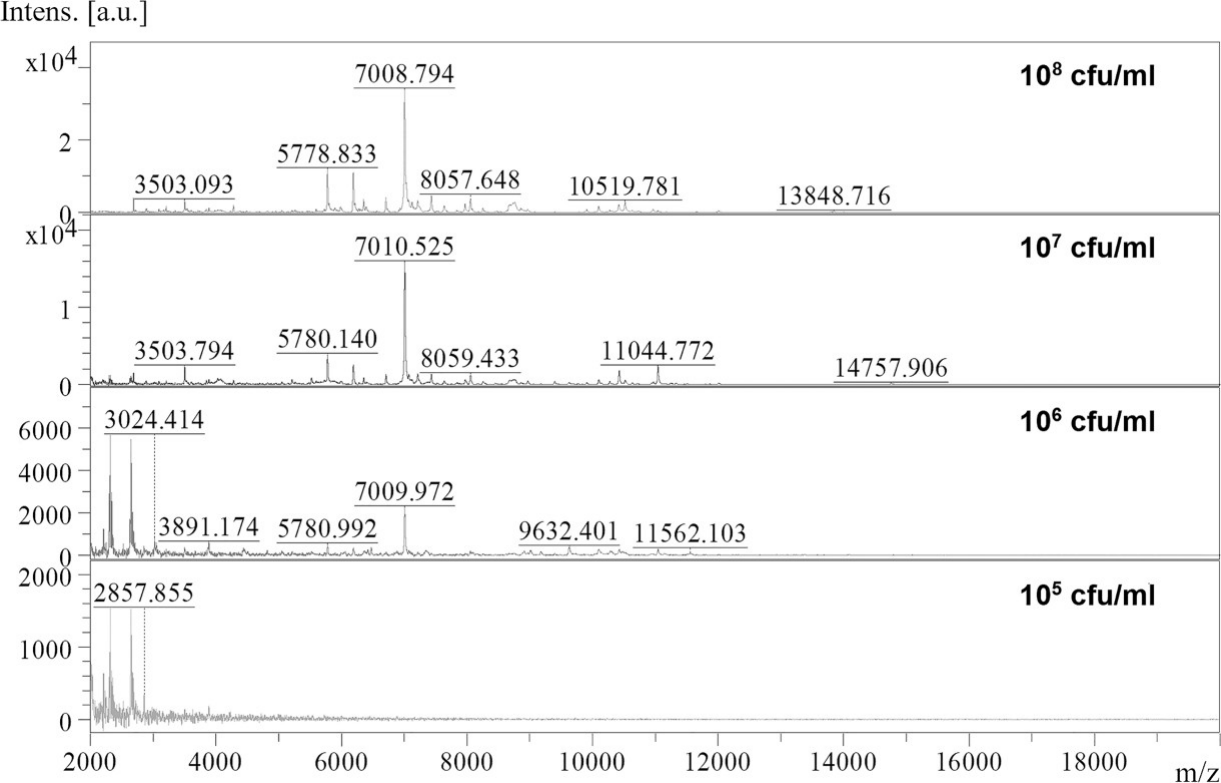
The protein mass spectra of *L*. *interrogans* at different bacterial concentration. X-axis represent Spectra at mass to charge ratio of 2000–20000, Y axis is peak intensity at arbitrary unit (a.u.).

The reproducibility of MSP from MALDI-TOF MS demonstrated by identification score of 3 *Leptospira* spp. (*L*. *interrogans*, *L*. *borgpetersenii* and *L*. *biflexa*) were higher than 2.0 after triplicate tested. The identification score of low passage *L*. *wolffii* strain H8, H9 and H16 revealed no statistically significant differences in two different growth media (p-value >0.05). According to sub-culture time, identification score of less than 6 sub-culture times (1–5 times) and that of more than 6 sub-culture times (6–10 times) have no statistically differences (p-value > 0.05). These results indicated the high reproducibility of *Leptospira* protein spectra generated by MALDI-TOF MS. The detection limit of MALDI-TOF MS method was demonstrated in 10-fold serial dilution of *L*. *interrogans* (MD08) protein spectra at 10^6^ CFU/ml (Fig 3).

The new protein spectra database of *Leptospira* spp. was created based on 116 *Leptospira* isolates of 15 references and 101 *Leptospira* isolates (including 100 pathogenic and 6 non-pathogenic group) as listed in Table 2. To validate this database, 97 *Leptospira* human isolates from Thailand and Laos were blindly analyzed based on profile matching to this *Leptospira* protein mass spectra database. The identification score of these isolates are shown in Table 4. The clinical isolates from Laos and Thailand were identified as *L*. *interrogans* (n = 84), *L*. *borgpetersenii* (n = 4), *L*. *weilii* (n = 7) and *L*. *kirschneri* (n = 1) with scores of 2.0–2.6. One isolates from Laos (No. UI14721) has score equal to 1.903 for *L*. *interrogans* best match and the second match (with score 1.702) was identified as the same species. The identification score evaluation of MALDI-TOF MS database demonstrated that 96 out of 97 (98.9%) correct identification at specie level.

**Table 4:** Identification score of blinded 97 *Leptospira* clinical isolates.

Identification score match with	Number of isolates	Identification score			Location (no. of isolates)	Year	Host	Source*
		Median	IQR	Range				
*L*. *interrogans*	84	2.4	2.34–2.51	2.015–2.635	Luang Namtha, Laos (n = 6)	2012–2014	Human	LOMWRU
					Salavan, Laos (n = 1)	2009	Human	LOMWRU
					Vientiane, Laos (n = 54)	2006–2008	Human	LOMWRU
					Udonthani, Thailand (n = 22)	2003–2006	Human	MORU
* *					Rayong, Thailand (n = 1)	2003	Human	MORU
*L*. *borgpetersenii*	4	2.28	2.23–2.34375	2.092–2.529	Mahasarakham, Thailand (n = 1)	2004	Human	MORU
* *					Vientiane, Laos (n = 3)	2007	Human	LOMWRU
*L*. *weilii*	7	2.2	2.1805–2.255	2.006–2.503	Luang Namtha, Laos (n = 4)	2012–2014	Human	LOMWRU
					Vientiane, Laos (n = 3)	2008–2009	Human	LOMWRU
*L*. *kirschneri*	1	2.473	-	-	Udonthani, Thailand (n = 1)	2003	Human	MORU
*Leptospira spp*.	1	1.903	-	-	Vientiane, Laos (n = 1)	2009	Human	LOMWRU

*Source

LOMWRU: Lao-Oxford-Mahosot Hospital-Wellcome Trust Research Unit, Lao PDR

MORU: Mahidol-Oxford Tropical Medicine Research Unit, Faculty of Tropical Medicine, Mahidol University, Thailand

### Biomarker identification for *Leptospira* spp and serovars

Protein mass spectra in mass range of 3,000 to 15,000 Da were analyzed by mathematical algorithms to define the protein peaks that could represent as biomarkers for leptospires. Discriminating peaks for pathogenic and intermediate leptospires were discovered by GA models. Eight peaks specifically presented in for pathogenic leptospires (Table 5) with 99.71% and 99.12% of RC and CV, respectively. The intermediate leptospires showed and 8 specific peaks (Table 6) with 100% of RC and CV. Peaks at m/z of 7006.70 for pathogenic and 6786.51 for intermediate leptospires demonstrated highest intensity and AUC value among peak list of the models. These indicated the best uniqueness and specificity of the peaks to pathogenic and intermediate leptospires, respectively.

**Table 5:** Peak statistics of 8 discriminating peaks for pathogenic leptospires included in the GA model.

m/z	Relative intensity (mean ± SD)		*P*-values^2^	AUC^3^
	Pathogenic	Non-Pathogenic^1^		
3502.55	7.70 ± 3.25	1.74 ± 1.41	0	0.98
5776.88	21.03 ± 11.99	1.12 ± 0.97	0	0.99
6352.39	8.91 ± 5.72	0.86 ± 0.25	0	0.98
7006.70	83.32 ± 41.54	4.63 ± 2.22	0	0.99^4^
7970.01	4.05 ± 2.15	0.55 ± 0.16	0	0.98
8734.95	3.81 ± 2.20	0.56 ± 0.22	0	0.94
10422.97	5.88 ± 4.56	1.05 ± 0.35	0	0.97
11040.17	8.74 ± 8.95	0.33 ± 0.15	0	0.99

^1^Non-Pathogenic includes intermediate and non-pathogenic (saprophytic) leptospires

^2^*P*-values are calculated based on Wilcoxon test

^3^AUC is area under the ROC curve

^4^The uniqueness and specificity peak to pathogenic leptospires indicated by the highest intensity and AUC value

**Table 6:** Peak statistics of 8 discriminating peaks for intermediate leptospires included in the GA model.

m/z	Relative intensity (mean ± SD)		*P*-values^2^	AUC^3^
	Intermediate	Non-Intermediate^1^		
3392.80	6.79 ± 1.71	1.77 ± 1.48	0	0.98
5958.35	10.48 ± 2.73	1.86 ± 4.65	0	0.98
6240.20	5.92 ± 5.54	3.53 ± 2.64	0	0.74
6547.87	3.27 ± 1.03	1.24 ± 0.39	0	0.95
6786.51	80.17 ± 19.21	1.28 ± 0.39	0	1.00^4^
7501.06	6.68 ± 1.62	1.63 ± 1.16	0	0.99
8512.63	6.67 ± 1.84	0.78 ± 0.34	0	1.00
8538.16	7.84 ± 2.44	0.72 ± 0.41	0	1.00

^1^Non-Intermediate includes pathogenic and non-pathogenic (saprophytic) leptospires

^2^*P*-values are calculated based on Wilcoxon test

^3^AUC is area under the ROC curve

^4^The uniqueness and specificity peak to intermediate leptospires indicated by the highest intensity and AUC value.

Ten specific peaks to discriminate among *L*. *interrogans*, *L*. *borgpetersenii*, and *L*. *kirschneri* were reported by the GA model with 99.2% and 96.1% of RC and CV, respectively. Each species of *Leptospira* showed a unique combination of peaks (Table 7). Four peaks at m/z of 3605.24, 5529.89, 8747.26, and 11260.81 were common peaks, which were observed in three *Leptospira* species (*L*. *interrogans*, *L*. *borgpetersenii*, and *L*. *kirschneri)*, but with differences in their relative intensities. Species-specific peaks were also detected. The presence of peaks at 6422.61 Da for *L*. *borgpetersenii* and peaks at 8084.81 Da for *L*. *kirschneri* was a unique characteristic of those two *Leptospira* species. Three peaks (6422.61 Da, 6924.67 Da, and 8084.81 Da) were not detected in *L*. *interrogans*. In addition, among the absent 3 peaks, lack of 6924.67 Da was specific to *L*. *interrogans*.

**Table 7:** Peaks for differentiating among 3 species of pathogenic *Leptospira* (*L*. *interrogens*, *L*. *kirschneri*, *and L*. *borgpetersenii*).

m/z	*Leptospira* species		
	*L*. *interrogans*	*L*. *kirschneri*	*L*. *borgpetersenii*
3605.24	**++**	**+**	**++**
5529.89	**++**	**+**	**+**
6422.61	**-**	**-**	**++**
6707.60	**++**	**++**	**-**
6924.67	**-**	**++**	**++**
8084.81	**-**	**++**	**-**
8260.69	**++**	**++**	**-**
8747.26	**++**	**+**	**+**
9924.00	**++**	**++**	**-**
11260.81	**++**	**++**	**+**

The demonstrated peaks are described in qualitative and quantitative data of relative intensity

The used symbols indicate

No peak: **-**

Present peak with lower intensity compared to the total average spectrum: **+**

Present peak with equal or higher intensity compared to the total average spectrum: **++**

Using the GA model, *L*. *interrogans* serovar-specific peaks were identified with 98.1% and 82.0% of RC and CV, respectively. The peak list was reported in Table 8. Regarding to the GA classification model which demonstrated as the best capacity for serovar discrimination, 19 peaks were picked to discriminate the 8 serovars of *L*. *interrogans* including Autumnalis, Bataviae, Conicola, Grippotyphosa, Medanensis, Pomona, Pyrogenes and Sejroe. Each serovar was characterized by the unique combination of peaks in both presence or absence and relative intensity level (Table 8). Seven out of the 8 serovars presented 17–18 peaks from all 19 peaks included in the model. The serovar Pomona showed all of the peaks. The serovar of Autumnalis was characterized by the absence of peak at m/z of 11325.41. The other serovars were demonstrated the 17 peaks as report in Table 8. Among the serovars with absence of 2 peaks, the serovar-specific pattern could be characterized by specific lacking of the peaks to all serovars but Autumnalis and Pomona. Particularly, the serovar Bataviae, Canicola, and Pyrogenes were characterized by the absence of the peak at 8619.54 Da, 10293.62 Da, and 5638.80 Da, respectively

**Table 8:** Peaks for differentiating among 8 serovars of *L*. *interrogans*.

m/z	Serovars of *L*. *interrogans*							
	Autumnalis	Bataviae	Canicola	Grippotyphosa	Medanensis	Pomona	Pyrogenes	Sejroe
3141.37	++	++	++	++	++	++	+	++
3210.83	++	+	++	+	++	+	+	++
4071.95	++	++	++	++	+	++	+	+
4283.23	++	++	+	+	++	++	++	++
4389.68	++	++	++	-	-	++	-	++
5638.80	++	++	++	++	+	++	-	++
6285.27	++	++	++	+	++	++	++	++
6946.20	++	+	++	+	++	++	++	++
7015.84	++	++	++	++	++	++	+	++
7221.33	++	++	++	++	++	++	++	++
7435.03	++	+	++	+	++	+	++	++
7852.87	++	++	++	++	++	++	++	+
8056.22	++	++	++	++	++	+	++	+
8619.54	++	-	++	++	++	+	++	++
8981.77	+	++	+	++	++	++	++	+
10293.62	+	+	-	++	+	++	++	+
10623.73	+	-	++	-	++	++	++	-
11061.85	+	++	-	++	-	++	+	+
11325.41	-	+	+	++	++	++	++	-

The demonstrated peaks are described in qualitative and quantitative data of relative intensity

The used symbols indicate: No peak: -

Present peak with lower intensity compared to the total average spectrum: +

Present peak with equal or higher intensity compared to the total average spectrum: ++

### Spiked *Leptospira* spp. in urine

**Fig 4:**
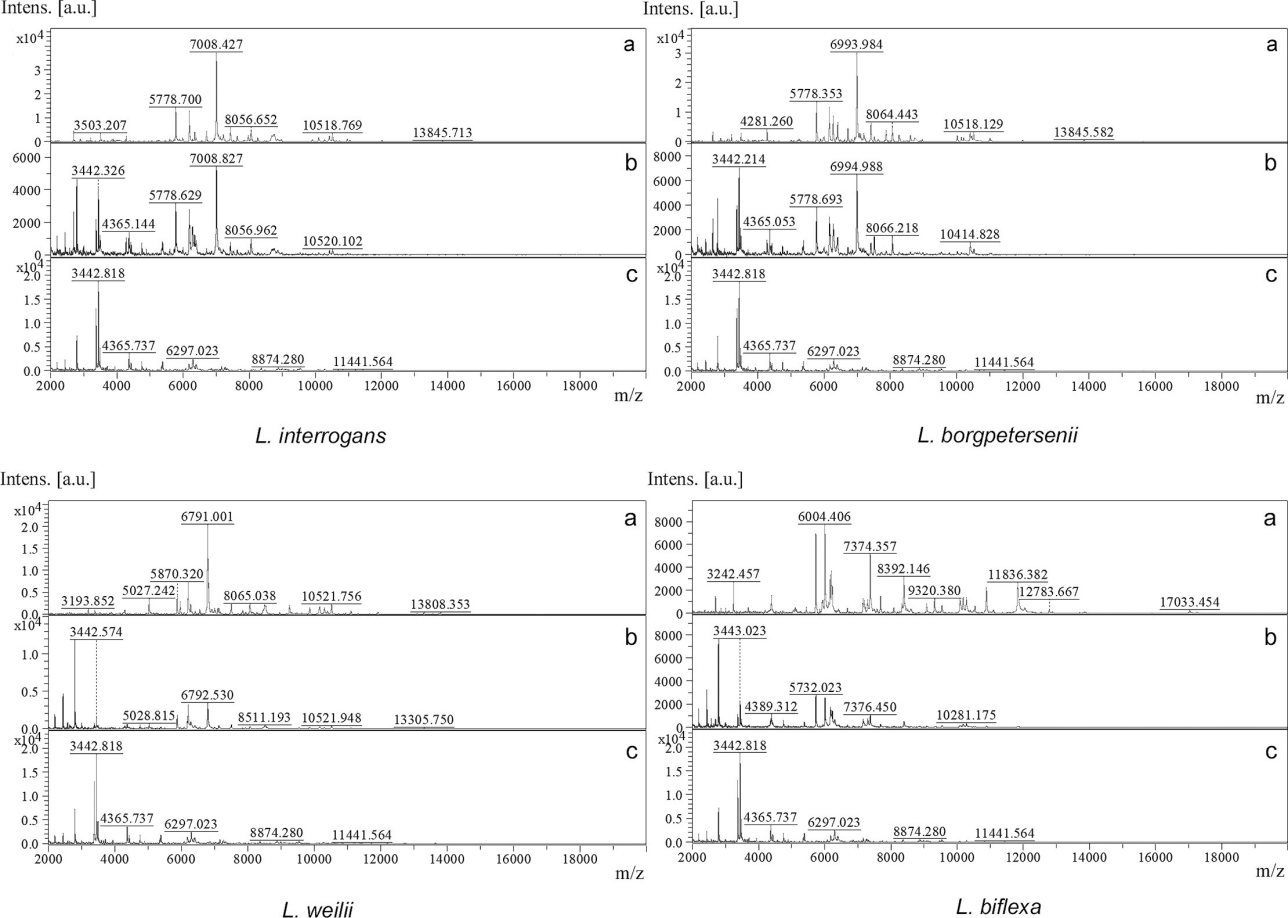
The protein profile matching of spiked leptospires in urine and media. The protein spectra of *L*. *interrogans*, *L*. *borgpetersenii*, *L*. *wolffii* and *L*. *biflexa* in culture media (a), spiked in urine (b). Urine without any leptospires used as control (c).

**Fig 5:**
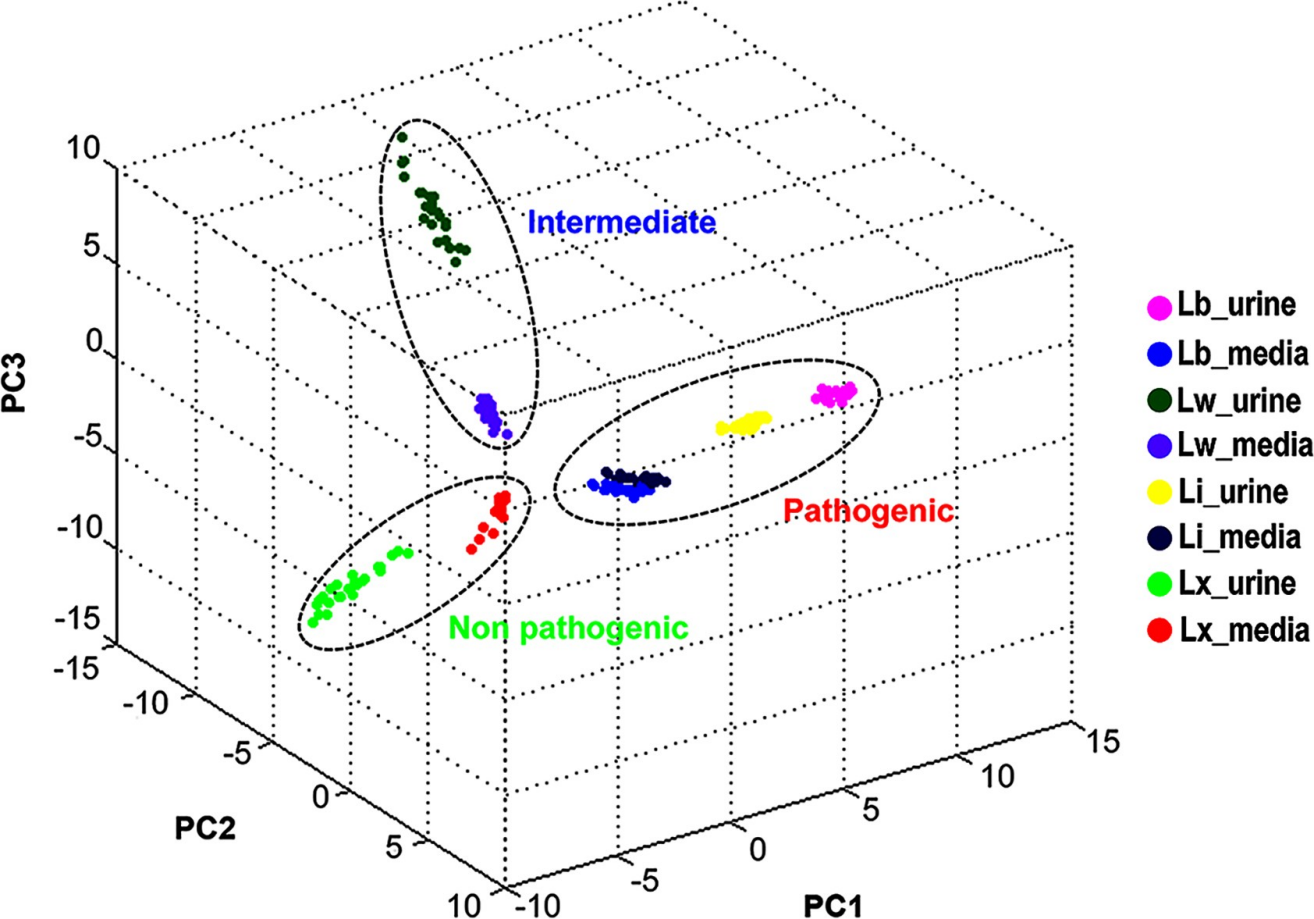
Principle component analysis (PCA) of protein spectra from spiked leptospires in urine and media. Each dot displayed a protein spectrum of each sample. Color dot represent *L*. *interrogans* (Li), *L*. *borgpetersenii* (Lb), *L*. *wolffii* (Lw) and *L*. *biflexa* (Lx) in media and urine. The clustering of pathogenic, intermediate and non-pathogenic group is demonstrated in dash line.

The protein spectra of 4 spiked *Leptospira* spp. (*L*. *interrogans*, *L*. *borgpetersenii*, *L*. *wolffii*, *L*.*biflexa*) demonstrated profile matching in both spiked urine and media (Fig 4). The small protein peak at m/z among 2,000–4,000 for example at m/z 3,442 were detected in protein profile of urine alone and in leptospires spiked urine. These peaks might be other ionized proteins in donor urine because they could not be detected in media. The identification score was high in both spiked leptospires in urine (range of 1.96–2.15) and in media (range of 2.0–2.5) while that of urine alone was unreliable (below 0.5). Using principle component analysis (PCA), 4 spiked *Leptospira* spp. in urine were located in the same cluster where each species represented; pathogenic group (*L*. *interrogans*, *L*. *borgpetersenii*), intermediate (*L*. *wolffii*) and non-pathogenic group (*L*. *biflexa*) as shown in Fig 5.

## Discussion

Whole cell MALDI-TOF MS of *Leptospira* was used to generate unique protein spectra or protein fingerprinting of 15 *Leptospira* spp. and clustered them according to their phylogeny. This study created the largest number of *Leptospira* protein database (116 protein spectra) that has been reported. The large protein database has strengthened the accuracy of *Leptospira* spp. MALDI-TOF MS identification with 98.9% correct identification of 96 *Leptospira* clinical isolates from different times and geographical areas of Thailand and Laos. One isolate with identification score below 2 possibly occurred because the protein spectra had low intensity. It was also possible that detection of proteins with relatively high molecular weight are more difficult to detect than protein in the low molecular weight range since this study used sinapinic acid matrix which ionized intact protein at high molecular weight [15].

MALDI-TOF MS method is simple, rapid and reliable for species identification but the method required bacterial culture which is of low sensitivity, laborious and time consuming. New culture agar called *Leptospira* Vanaporn Wuthiekanun (LVW) for *Leptospira* could reduce the isolation time but they still required a week for culture [14, 16]. Urine is a non-invasive, in comparison with serum or blood, which might be of interest for diagnosis of leptospirosis. Our results using spiked 10^8^ cfu/ml leptospires in urine are suggesting that MALDI-TOF MS method may be able to identify *Leptospira* in urine. However, there are limitations. We lacked urine from leptospirosis patients and further investigation on detection limit of *Leptospira* in urine is required. The lowest amount of *Leptospira* which could generate the protein spectra by MALDI-TOF MS was 10^6^ cfu/ml of culture media which is comparable to two reports [8, 9]. However, the number of *Leptospira* in mammal urine is varied. A meta-analysis on the quantity of *Leptospira* in urine of infected animals and human in Ecuador has reported that rats excreted amount of *Leptospira* in urine with median of 5.7 x 10^6^ cells/ml while large mammals excreted and shed 5.1 × 10^8^ to 1.3 × 10^9^ cells *Leptospira* per day [17]. Moreover, protein spectra of human and animal urine need identification in order to clearly define disease-associated biomarker. The standardize method for detection of *Leptospira* in human or animal urine needed to be optimized and validated.

The major benefit from large *Leptospira* protein spectra database was that the database can be used as training set for machine learning and making a decision based on the computational mathematic analysis which demonstrated good classification in phylogeny. The specific peak at m/z of 6786.51 and 7006.70 were consistently found in intermediate and pathogenic *Leptorspira* respectively. These 2 specific peaks could be used as biomarker to differentiate between those 2 groups. Moreover, three *Leptospira* spp. (*L*. *interrogans*, *L*. *kirschneri*, and *L*. *borgpetersenii*) which are most found species from rodent in Thailand, Laos and Cambodia [18] could present the differentiating peaks among each other. There were few studies [8–10] reporting the difficulty to differentiate between *L*. *interrogans* and *L*. *kirschneri* using MALDI-TOF MS. Our data revealed that two peak at m/z of 6924.67 and 8084.81 were presented in *L*. *kirschneri* but absent in *L*. *interrogans*.

Serotyping is useful in one health epidemiological studies of Leptospirosis. The attempt of serotype differentiation by MALDI-TOF MS was examined and indicated up to 19 peaks among 8 serotypes (serovars) of *L*. *interrogans*. However, the low number of individual serovars used as training set here may reduce the result accuracy. In addition, serovar diversity of *Leptospira* is based upon variation of lipopolysaccharide (LPS) at the bacterial outer membrane [19]. LPS structure consists of a lipid and a polysaccharide of O-antigen, outer core and inner core. Those components may not be ionized using MALDI-TOF MS settings adapted for bacterial protein detection. Besides, *Leptospira* isolates in Thailand has been reported as unpredicted serovars by microscopic agglutination test (MAT) [20].

In conclusion, our whole cell MALDI-TOF MS method can cluster *Leptospira* spp. according to their phylogeny and identify the large scale cultured leptospiral strains at the species level based on their protein profile with highly reproducibility. The biomarkers based on protein profile could be used for identification the common pathogenic species *L*. *interrogans*, *L*. *kirschneri*, and *L*. *borgpetersenii* of human isolates in endemic areas like Thailand and Laos PDR. Therefore, whole cell MALDI-TOF MS method is a simple, faster and reliable tool for confirmation of leptospiral species.
